# Supplementary material for: Pathogen detection in acellular cerebrospinal fluid: diagnostic insights from a pediatric cohort in Colombia
Source: Microbiol Spectr. 2025 Nov 24;14(1):e02122-25. doi: 10.1128/spectrum.02122-25 (PMC12772229; doi:10.1128/spectrum.02122-25)
Supplement: Table S1 — Clinical profile, detected pathogens, and retrospective adjudication of 21 pediatric patients with positive FA-M/E results. [file spectrum.02122-25-s0001.docx]

| **Case #** | **Sex** | **Age (months)** | **LP Indication / Chief Complaint** | **Fever (Y/N)** | **Seizures (Y/N)** | **Neck Stiffness (Y/N)** | **GCS** | **Prior Abx Use (Y/N/U)** | **Pathogen 1 (FA-M/E)** | **Pathogen 2 (FA-M/E)** | **Hospital Stay (days)** | **Discharge Diagnosis** | **Retrospective Clinical Adjudication (RCA)** |
| --- | --- | --- | --- | --- | --- | --- | --- | --- | --- | --- | --- | --- | --- |
| 1 | F | 8 | Respiratory Distress | N | N | N | 15/15 | N | Enterovirus | N/D | 7 | Viral Meningitis | Probable Infection |
| 2 | M | 1 | Fever | Y | N | N | 15/15 | N | HHV-6 | N/D | 3 | Neonatal Sepsis (other bacteria) | Possible Reactivation/Latency |
| 3 | F | 1 | Fever | Y | N | N | 15/15 | N | HHV-6 | N/D | 3 | Neonatal Sepsis (other bacteria) | Possible Reactivation/Latency |
| 4 | F | 2 | Fever | Y | N | N | 15/15 | N | N. meningitidis | N/D | 10 | N. meningitidis Meningitis | Definite Infection |
| 5 | F | 5 | Altered Consciousness | N | N | N | 15/15 | N | E. coli K1 | N/D | 12 | Epilepsy, Global Neurodevelopmental Delay | Indeterminate / Colonization |
| 6 | M | 10 | Fever | Y | N | N | 15/15 | N | S. agalactiae | HHV-6 (11) | 7 | Neonatal Sepsis (other bacteria) | Definite Infection (Bacterial) |
| 7 | F | 16 | Irritability | Y | N | N | 15/15 | N | Enterovirus | N/D | 5 | Enteroviral Meningitis | Probable Infection |
| 8 | F | 10 | Fever | Y | Y | N | 15/15 | N | HSV-2 | N/D | 4 | HSV-2 Neuroinfection | Definite Infection |
| 9 | F | 10 | Fever | N | N | N | 15/15 | N | E. coli K1 | Enterovirus (8) | 10 | Neonatal Sepsis (other bacteria) | Probable Infection (Mixed) |
| 10 | M | 16 | Fever | Y | N | Y | 15/15 | Y | Enterovirus | N/D | 43 | Other Bacterial Meningitis | Probable Infection (Viral) |
| 11 | F | 17 | Fever | Y | N | N | 15/15 | U | Enterovirus | N/D | 6 | Other Bacterial Pneumonias | Probable Infection (Viral) |
| 12 | M | 1 | Fever | Y | N | N | 15/15 | N | Enterovirus | N/D | 5 | Unspecified Neonatal Bacterial Sepsis | Probable Infection (Viral) |
| 13 | M | 7 | Weakness, Eye Rolling | N | N | N | 15/15 | N | HHV-6 | N/D | 18 | Aseptic Meningoencephalitis due to HHV-6 | Probable Infection |
| 14 | F | 12 | Fever | Y | N | N | 15/15 | N | Enterovirus | N/D | 3 | Neonatal Sepsis (anaerobes) | Probable Infection (Viral) |
| 15 | M | 40 | Eye Deviation | N | N | N | 15/15 | Y | S. pneumoniae | N/D | 13 | Secondary Pneumococcal Neuroinfection | Definite Infection |
| 16 | M | 13 | Oliguria, Resp. Distress | N | N | N | 15/15 | Y | Enterovirus | N/D | 7 | Respiratory Distress Syndrome: Pneumonia | Possible Infection / Bystander |
| 17 | M | 15 | Fever and Seizures | Y | Y | N | 15/15 | Y | E. coli K1 | N/D | 11 | Neonatal Bacterial Sepsis | Definite Infection |
| 18 | M | 156 | Nephrotic Syndrome | N | N | N | 15/15 | Y | CMV | N/D | 10 | Other CMV diseases | Probable Reactivation |
| 19 | F | 24 | Poor Feeding | Y | N | N | 14/15 | Y | HHV-6 | N/D | 19 | Meningitis (other infectious diseases) | Probable Infection |
| 20 | F | 20 | Fever | Y | N | N | 15/15 | N | Enterovirus | HHV-6 (11) | 6 | Late Neonatal Sepsis | Probable Infection (Viral) |
| 21 | M | 5 | Seizure | Y | Y | N | 15/15 | N | HHV-6 | N/D | 2 | Roseola due to HHV-6 | Probable Infection |

**Supplementary Table S1. Clinical Profile, Detected Pathogens, and Retrospective Adjudication of 21 Pediatric Patients with Positive FA-M/E Results.** Detailed clinical, demographic, and etiological profile for the 21 pediatric patients enrolled in the Cartagena, Colombia cohort who tested positive for a Central Nervous System (CNS) pathogen using the FilmArray® Meningitis/Encephalitis (FA-M/E) panel. These patients were included based on clinical suspicion requiring lumbar puncture (LP), generally defined as fever plus one or more neurological signs (such as seizures, altered consciousness, focal neurological deficit, or meningeal signs), or neonates presenting with fever without source, according to institutional protocol.The table includes demographic data (Sex, Age), clinical parameters at presentation (Chief Complaint/LP Indication, GCS, presence of Fever, Seizures, and Neck Stiffness), history of Prior Antibiotic Use (Abx Use), the specific pathogen(s) detected by the FA-M/E panel (Pathogen 1 and Pathogen 2), Length of Hospital Stay (days), and the ultimate Discharge Diagnosis. A critical feature highlighted by this cohort is that 100\% of these 21 FA-M/E positive samples exhibited a complete absence of pleocytosis (acellular CSF), challenging traditional diagnostic paradigms. **Abbreviations:** F=Female, M=Male, GCS=Glasgow Coma Scale, Abx=Antibiotics, N/D=Not Detected. Y=Yes, N=No, U=Unknown. FA-M/E=FilmArray® Meningitis/Encephalitis panel. LP=Lumbar Puncture. RCA=Retrospective Clinical Adjudication.
